# The Use of Service Dogs for People With Physical Disabilities in Japan in Accordance With the Act on Assistance Dogs for Physically Disabled Persons

**DOI:** 10.3389/fvets.2019.00198

**Published:** 2019-06-21

**Authors:** Tomoko Takayanagi, Mariko Yamamoto

**Affiliations:** ^1^Japan Service Dog Association, Kanagawa, Japan; ^2^Department of Animal Sciences, Teikyo University of Science, Yamanashi, Japan

**Keywords:** assistance dog, Japan, service dog, guide dog, hearing dog, people with disabilities, act on assistance dogs

## Abstract

Japan learnt how to promote assistance dogs effectively by deliberating the issues and challenges that surrounded assistance dogs in the USA and Europe and the Act on Assistance Dogs for Physically Disabled Persons was issued in 2002. The aim of this paper is to provide information that may be useful for countries and areas that are seeking ways to regulate assistance dogs, especially in the context of the global problem in which dogs are falsely claimed to assist their partners. First, there is a description of the process through which Japan, where pet dogs have not been accepted in society, established the Act, which overcame the shortcomings of the previous situation. Second, it is shown the ways in which people living with assistance dogs have gained the right to have their dogs accompany them in public. Third, the current challenges faced by people with assistance dogs are documented. Finally, pictures of an example of an assistance dog certificate and of an assistance dog sign reveal how far the regulation of assistance dogs is achieved in Japan.

## Introduction

**Figure 1:**
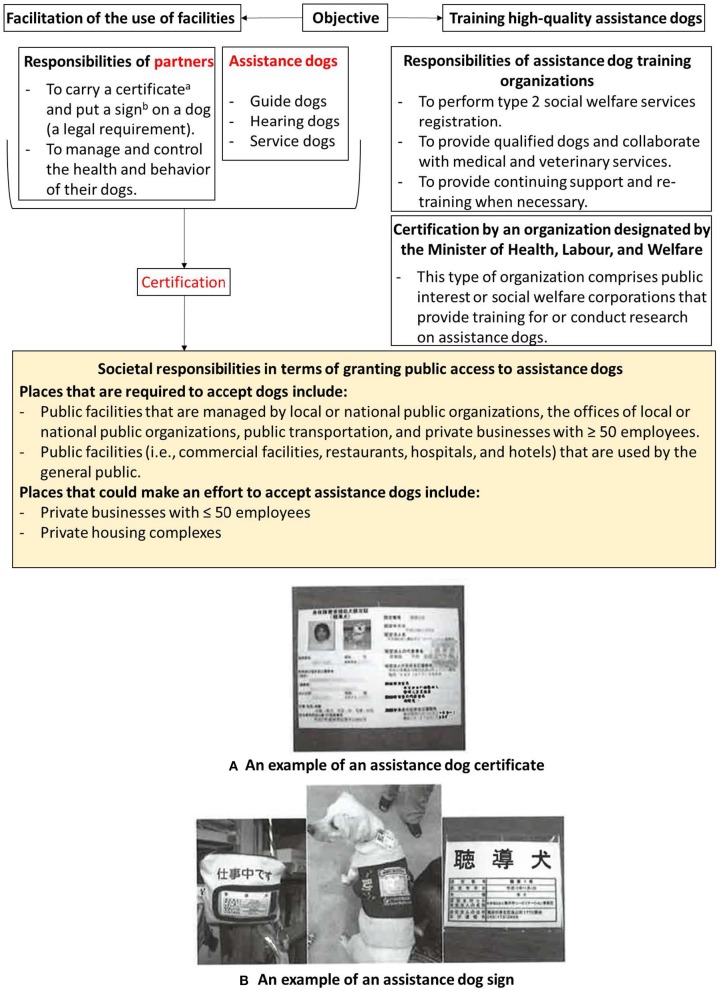
Overview of the act on assistance dogs for physically disabled persons. **(A)** The assistance dog certificate shows photographs of the dog service partner and his or her assistance dog; the name, date of birth, address, and contact information of the service dog partner; the name, date of birth, breed, hair color, hair texture, and registration number of the dog, in accordance with the rabies prevention law; the certification number and date; the name, address, and contact information of the designated corporation; and the name, address and contact information of the training organization. **(B)** The assistance dog sign indicates (from left to right) a guide, service, or hearing dog sign, with certification number and certified date; the breed of the dog; and the name, address, and contact information of the designated corporation that approved the certification.

The first guide dog was domestically trained in Japan in 1957, 60 years ago (1). Training for service and hearing dogs began in the 1990s and 1981, respectively (2, 3). Japan adopted the philosophy of training dogs and using them to assist people with disabilities from the USA and other European countries. However, initially it was difficult for assistance dogs to gain acceptance in Japan because historically, people in that country had a different relationship with dogs compared to people in the West. Dogs had not been trained to fit in human society in Japan (4). Generally, people used them as watch dogs; placing them on a leash outside the house. The importance of training friendly, socialized dogs was recognized only relatively recently, in the late 1990s (4). Therefore, there were many obstacles that were unique to Japan that had to be overcome before assistance dogs could be used in Japan. Japan learnt how to promote assistance dogs effectively by deliberating the issues and challenges that surrounded assistance dogs in the USA and Europe. This is evident through the establishment of the Act on Assistance Dogs for Physically Disabled Persons in Japan on May 22, 2002 (5), whose stated goal was to facilitate the growth of the quality of assistance dogs and the use of public facilities for people with physical disabilities, with a view to contributing to the independence and social participation of such individuals. The act also stipulates the responsibilities of society, assistance dogs training organizations, certifying organizations, and assistance dog partners (Figure 1).

Similar to Japan, some countries and states, such as Taiwan and Queensland, Australia, respectively, have established laws that define the standards and certification of assistance dogs (6–8). However, such laws do not exist in most countries. The US and some European countries recognize only the right of people with disabilities to have their assistance dogs accompany them in places where pet dogs are not allowed under the law (9, 10). In countries where regulations regarding assistance dogs or consensus regarding best practices on raising assistance dogs (11–13) do not exist, falsely claimed assistance dogs and assistance dogs of poor quality have become a serious problem (14, 15). This paper describes the process of Japan's establishment of the Act on Assistance Dogs for Physically Disabled Persons. The establishment of this Act overcame the shortcomings of previous situations, and people living with assistance dogs have gained the right to have their dogs accompany them in public. However, these people still face challenges. The aim of this paper is to provide information that may be useful for countries and areas that are seeking ways to regulate assistance dogs.

Prior to the issue of the Act on Assistance Dogs for Physically Disabled Persons, assistance dogs other than guide dogs were treated in the same way as pet dogs. Moreover, assistance dogs were not allowed in public. The public access of guide dogs and their partners was protected under the official notices of several government ministries, including the Ministry of Transport, the Ministry of Construction (currently merged in the Ministry of Land, Infrastructure, Transport and Tourism), the Ministry of the Environment, and the Ministry of Health and Welfare (16). However, these official notices did not have the force of law, and some people with guide dogs were still prevented from entering facilities (16). The Act on Assistance Dogs for Physically Disabled Persons has greatly improved this situation.

Before the act was established, pet dogs were not accepted (4). Hence, assistance dog owners lobbied to gain the right of public access. They submitted strong proposals for fulfilling their responsibilities for ensuring public health by preventing their dogs from spreading zoonotic diseases, destroying things, injuring, and threatening others. Hence, the process of certifying assistance dogs and their human partners was conducted in detail, which is explained later in this paper. People believed that in the certifying process, raising the hurdles would be more difficult than lowering them; therefore, they were set at a high level.

The act guarantees the participation of people with disabilities in Japanese society by specifying that facilities are not permitted to deny access to people who are accompanied by certified guide, service, and hearing dogs. The act can be thought of as disability discrimination legislation. It precedes the Act for Eliminating Discrimination Against Persons with Disabilities Act that was enforced in Japan in April 2016. According to the Act on Assistance Dogs for Physically Disabled Persons in Japan, a service and hearing dog training organization is required to register as a type 2 social welfare services at a local prefectural government. Currently, there are 26 training organizations for service dogs and 21 for hearing dogs, 14 of which train both types of dog (17). Nevertheless, a limited number of training organizations actually provide service and hearing dogs annually. Guide dogs have a longer history than the other two types of dog. Accordingly, they are trained by organizations that are designated by the National Public Safety Commission, of which there are currently 11 nationally (18).

The law also requires that organizations that are designated by the Minister of Health, Labor, and Welfare administer certification examinations to people with disabilities and their service or hearing dogs. Certification results if the examination can confirm that the assistance dog partners are in a position to assume responsibility for the health, hygiene, and behavior of their assistance dog when encountering society. There are seven organizations that certify service dogs and six organizations that certify hearing dogs. Among them are three organizations that also train service dogs and hearing dogs. The certification process does not apply to guide dogs. Appropriate training is given to guide dogs and their partners through the organizations designated by the National Public Safety Commission. After certification, they must carry their certificate on them at all times. The assistance dogs are also required to wear an assistance dog sign when exercising their right to access public and private spaces (Figure 1).

## Definition and the Present Circumstances of Assistance Dogs used to Assist People with Physical Disabilities in Japan

The Act on Assistance Dogs for Physically Disabled Persons in Japan defines assistance dogs as dogs that are trained with the aim of promoting the independence and social participation of people with disabilities. Three types of assistance dogs in Japan (guide dogs, service dogs, and hearing dogs) are used to aid people with visual impairment, impaired mobility, and hearing impairment, respectively. The disability of the assisted person is indicated on a physical disability certificate. The Japanese certificate adheres to the stipulations cited in the Law for the Welfare of People with Physical Disabilities. It is issued by a prefectural governor provided that a governor-certified medical doctor has determined that a person has met the diagnostic criteria and is thus eligible to be certified. Therefore, the certificate functions in a similar way to a passport because it proves that the certificate holder is entitled to receive several social welfare benefits, including medical expense subsidies, prosthetic devices, housing renovation costs to improve his or her living environment, a reduction in income tax, and discounted public transportation. The physical disability certificate identifies those who have a disability and those who are eligible to receive social welfare benefits. The Act on Assistance Dogs for Physically Disabled Persons is also based on this law, and it defines those who are eligible to own an assistance dog. The physical disability certificate supports the act. Some people with false assistance dogs may also claim that they have a disability even though they do not. The physical disability certificate can prevent such situations from occurring.

Nine hundred and forty-one working guide dogs were registered in Japan in March 2018; a considerable decline from 1,070 in 2011. Sixty-six service dogs and 67 hearing dogs were registered in the country in January 2019 (19). While service dogs in the USA and certain countries in Europe are trained to assist people with mental illness and autism, the provision of Japanese service dogs is only applicable to people with physical disabilities. Unlike guide and hearing dogs, the extent to which people have impaired mobility varies considerably, meaning that their needs also vary. Accordingly, rehabilitation professionals are tasked to determine the tasks for which the dogs must be trained, to match service dogs with partners, and advise on suitable ways in which partners can take care of and control their dogs. The current practices and use of service dogs in Japan are now covered.

### Service Dog Uses

Classification of the disabilities of 70 dog assistance partners was shown to include neuromuscular disease (excluding stroke) (26%), cervical spine injuries (17%), spinal cord injuries (12%), cerebral infarction sequela (10%), and cerebral palsy (10%) in a recent study (20). The average age of the dog assistance partners ranged from 46 to 49 years, three quarters (71%) of whom were grade 1 certificate holders and 19% of whom were grade 2 holders. This indicates that 90% of the study subjects had severe mobility impairments. Classification of grade 1 or 2 in mobility disabilities according to the Law for the Welfare of People with Physical Disabilities is:

#### Grade 1 Impairment

Grade 1 impairment includes (1) upper limb impairment, i.e., total loss of function on both upper limbs or amputation above the wrist on both upper limbs; (2) lower limb impairment, i.e., total loss of function on both lower limbs or transfemoral (above-knee) amputation on both lower limbs; and (3) trunk impairment, i.e., the absence of balance while sitting.

#### Grade 2 Impairment

Grade 2 impairment includes (1) upper limb impairment, i.e., severe loss of function on both upper limbs, the amputation of five fingers on both hands, amputation above half-length of the humorous on one of the upper limbs, or total loss of function on one of the upper limbs; (2) lower limb impairment, i.e., the severe loss of function on both lower limbs or amputation above half-length of the lower thigh on both lower limbs; and (3) trunk impairment, i.e., difficulty sitting or standing, or difficulty standing up.

The accreditation of assistance dog-partner teams presupposes that the partners will take full responsibility for their assistance dogs when exercising their legal right to public access and that their dogs will not inconvenience others. Thus, each partner is evaluated for his or her suitability when acquiring an assistance dog. The evaluation is similar to that of a test for a driver's license. A thorough evaluation must be conducted by a group of professionals, including a rehabilitation physician, physiotherapist, and occupational therapist; to ensure various perspectives to determine whether or not the partner's quality of life and self-reliance would be improved by assistance from a service dog. For example, service dogs are particularly effective in improving the self-reliance of people with C6 cervical cord spinal injuries, and who constitute a large proportion of the population needing service dog partners in Japan. These dogs enable their partners to go out by themselves supported by equipment that has specifically been prepared by occupational therapists to cater to their needs. However, it has been found that the use of service dogs for partners with C5 injuries or above is unlikely to lead to enhanced independence. A secondary consideration is that partners with C5 injuries require substantial assistance in managing and caring for their service dogs.

**Figure 2:**
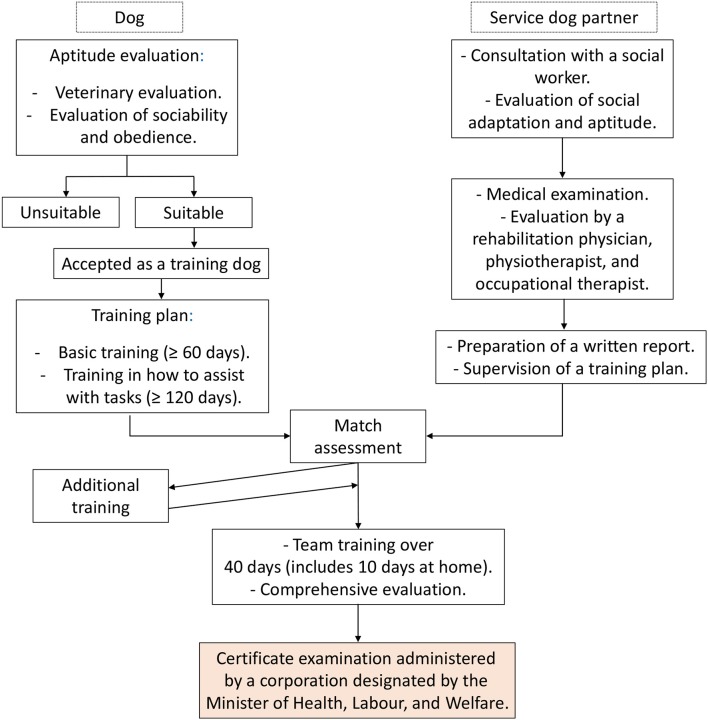
A flow chart of the certification procedure at the Yokohama City Rehabilitation Center, Kanagawa, Japan. The upper left side of the flow chart depicts the training of a service dog, while the upper right side details the evaluation of a person in terms of eligibility for being a service dog partner. Team training only begins once the person is deemed to be eligible. The team is certified once it has passed a final certificate examination that is administered by a corporation that has been designated by the Minister of Health, Labor, and Welfare.

The effective use of service dogs has been attributed to service dog partners with progressive diseases, including neuromuscular diseases, i.e., multiple sclerosis; severe myasthenia gravis; Becker muscular dystrophy; and spinal muscular atrophy. Nevertheless, ongoing disease monitoring must be performed for this patient group as, for example, it is thought that service dog partners experience a detrimental rather than a beneficial effect when there is rapid disease progression, i.e., occurring within a year or over several months. Once a service dog and partner have been paired, they receive team training at the partner's home or training center (Figure 2).

Public training extends to visits to stores, various amenities, and public transportation. The training is supervised by medical professionals because it must not exacerbate the service dog partner's medical condition. Service dogs are lent by training organizations free of charge to partners. However, it is the partner's responsibility to manage the daily health, sanitary, and general behavior of his or her service dog. This can pose a considerable strain for a partner if his or her medical condition is unstable.

### Eligibility for Certification as a Service Dog Partner

Similar to the way in which it is necessary to evaluate the eligibility of an applicant for a driver's license or his or her ability to handle an electric wheelchair, an applicant wanting a service dog must be assessed for his or her ability to assume responsibility for that dog. This is achieved by undertaking and passing an assistance dog certificate examination. A flow chart of the certification procedure at the Yokohama City Rehabilitation Center details the steps that need to be taken in this regard (Figure 2). Consultations are offered out at the Yokohama City, Nagoya City, Chiba Prefecture, and Hyogo Prefecture Rehabilitation Centers at which potential applicants are advised on how to become a service dog partner. Partners and their service dogs receive partial training, and certification is performed by a corporation that has been designated by the Minister of Health, Labor, and Welfare.

The ability of the accompanying service dog to comprehend and uphold the responsibilities in public is evaluated by a social worker or similarly qualified medical professional during the consultation process. If it is determined that the prospective partner lacks the intellectual, mental, developmental, or necessary brain capacity to provide proper and continuous care to the dog and/or optimally manage his or her health, the candidate will be informed that he or she is ineligible to partner with the service dog, even if the dog could be of help in certain situations. Above all, it is believed that the consultation service represents an opportunity to identify issues that might impede the quality of life of a prospective partner and determine what he or she expects from the service dog. This also provides the prospective partner with an opportunity to obtain useful information about related social welfare services and equipment, as well as facilitate an assessment of his or her capacity for rehabilitation. Further information can be obtained in this regard, if required. The consultation process represents an opportunity to help people improve their quality of life by offering them opportunities that will facilitate their independence and social participation that include, but are not limited to, service dogs.

### The Consultation Process, Service Dog Lending, and Follow-Up Support

Service dogs act as an important aid to partners. However, the process of acquiring a service dog is different to that involved in obtaining a prosthetic device. A prosthetic device is often recommended for people with disabilities by a rehabilitation specialist. Conversely, people with disability often request a service dog because rehabilitation specialists are not always familiar with this concept. People learn about service dogs through television, newspapers, and demonstrations at events held by training organizations. Having requested a service dog, the evaluation process is commenced by training organizations and professionals at designated rehabilitation centers. After matching a prospective partner with a potential service dog in accordance with a written opinion from a rehabilitation specialist, the team training commences. Following the completion and approval of a certificate examination, the dog is officially certified as a service dog, and the person and dog begin to live together. It is the responsibility of the training organizations to continue to provide assistance to the service dog partners. It is also their obligation to provide re-training to partners and their service dogs in the event of a change in circumstances, i.e., the progression of disability, or if the environment of the partner changes.

### Certification Procedures for Guide Dogs and Hearing Dogs

Public access by people living with guide dogs and hearing dogs is protected under the same act. The procedure for certifying hearing dogs and their human partners is the same as the process for certifying service dogs and their owners. Exceptions to the procedure for certifying hearing dogs and their partners are human health professionals, such as speech therapists. Guide dogs have a longer history in Japan than service dogs and hearing dogs do. Therefore, guide dogs are certified by a slightly different procedure than those used for service dogs and hearing dogs. Guide dogs are trained by an organization designated by the National Public Safety Commission, and the same organization also certifies the dogs that they train. Otherwise, the procedures and responsibilities of the training organizations and the partners are the same for hearing dogs and service dogs.

## Discussion

The roles of assistance dogs have expanded in the US and European countries (13), where regulations and laws for the training and certification of assistance dogs usually do not exist. Hence, it is possible for people who believe in the potential ability of dogs to create their own assistance dogs. However, the lack of regulation also facilitates people who falsely claim that their pet dogs are assistance dogs. Incidents of bites by falsely claimed assistance dogs and assistance dogs with inadequate temperaments have been also reported, and death and serious injuries have occurred (21, 22). Therefore, it is essential to regulate assistance dogs to protect the right of public access for people living with adequately trained assistance dogs and to maintain the safety of the public. It is also necessary to protect the welfare of dogs so that those with inadequate temperaments are stressed by stimulations in public or the tasks that are expected of them. The Act on Assistance Dogs for Physically Disabled Persons allows assistance dog partners to confidently accompany their assistance dogs in public. In addition, members of the public are able to accept assistance dogs because the act stipulates the responsibilities of assistance dog training organizations, certifying organizations, assistance dog partners, and society.

Although the act has promoted the field of assistance dogs in Japan, some challenges remain. First, the act applies only to guide dogs for people with visual impairments, hearing dogs for people with hearing impairments, and mobility service dogs for people with mobility impairments. Other types of service dogs, such as those for people with psychiatric disabilities and children with autism, are not covered by the act. The need for such dogs was not recognized when the act was established. Moreover, only guide dog partners, hearing dog partners, and mobility service dog partners lobbied to establish the act. Moreover, the act was established based on the major premise that the partners of assistance dogs must be responsible for them in public. Therefore, people who are unable to fulfill these responsibilities cannot be an assistance dog partner. An example is children with disabilities. Assistance dogs that are not covered by the act could be included if their partners' needs are recognized in the future. Second, assistance dog partners are still prevented from using facilities even though the act was established 17 years ago. The reason is that the act has not gained enough public recognition, and there is no punishment for people or facilities that deny entrance to assistance dogs and their partners. Most people have not encountered assistance dogs and their human partners in public because their number is limited in Japan. The result is that many people do not recognize the act, and they do not know how to interact with a person with an assistance dog. Therefore, people with assistance dogs are still forced to live restricted lives (23), which is the main challenge that needs to be addressed. Lastly, some assistance dog training organizations also certify them. Hence, not all assistance dogs are certified by a third party, which hinders objectivity in assessing and maintaining the quality of assistance dogs. However, the Ministry of Health, Labor, and Welfare is now revising the act to resolve this problem.

## Conclusion

The definition of “assistance dog” is clearly explained on the Act on Assistance Dogs for Physically Disabled Persons, and the process of training and certification is based on this law. Challenges with verifying the authenticity of assistance dogs, while experienced in other countries, are generally not encountered in Japan as it is relatively easy to verify this by asking to see the dog's sign and the certificate. Therefore, it can be said that the Act on Assistance Dogs for Physically Disabled Persons in Japan has created a strong foundation for assistance dog partners to confidently accompany their assistance dogs in public, and people are able to accept assistance dogs with peace of mind. However, some remaining challenges need to be resolved.

## Data Availability

No datasets were generated or analyzed for this study.

## Author Contributions

TT and MY, the two authors, equally contributed to write the mini review.

### Conflict of Interest Statement

The authors declare that the research was conducted in the absence of any commercial or financial relationships that could be construed as a potential conflict of interest.
